# Effect of strength training on functional outcomes and strength in patients with polyneuropathy: A scoping review

**DOI:** 10.3389/fphys.2023.1158039

**Published:** 2023-04-06

**Authors:** Britt Stævnsbo Pedersen, Louise Sloth Kodal, Anna Bundgaard Kaalund, Sonja Holm-Yildiz, Mette Merete Pedersen, Tina Dysgaard

**Affiliations:** ^1^ Copenhagen Neuromuscular Center, Department of Neurology, Rigshospitalet, Copenhagen University Hospital, Copenhagen, Denmark; ^2^ Department of Clinical Research and Physical Medicine and Rehabilitation Research Copenhagen (PMR-C), Copenhagen University Hospital Amager and Hvidovre, Hvidovre, Denmark; ^3^ Department of Clinical Medicine, Faculty of Health and Medical Sciences, University of Copenhagen, Copenhagen, Denmark

**Keywords:** polyneuropathy, strength training, resistance training, immune-mediated polyneuropathy, diabetic polyneuropathy, hereditary polyneuropathy, muscle strength, functional outcomes

## Abstract

**Introduction:** Polyneuropathy (PNP) is a chronic progressive disease that over time can lead to damage of sensory, motor and/or autonomic peripheral nerves. Symptoms vary from predominantly sensory to severe sensorimotor affection both proximally and distally. This can result in considerable functional impairments that affect activities of daily living. In other neurological patients, strength training has shown to improve strength and functional outcomes. Since medical treatment only exists for very few percentages of the underlying causes it is obvious to consider if strength training could be a potential treatment for functional impairments. To date little is known on the effect of strength training in patients with PNP.

**Aim:** The aim of this scoping review was to summarize research on strength training and outcomes on physical function in patients with PNP.

**Methods:** We systematically searched five data bases; Pubmed, Embase, Cinahl, Cochrane library and Web of science. Studies on strength training (load ≥70% of 1RM) in patients with PNP were included. The search was carried out in November 2022.

**Results:** 362 articles were screened by title and abstract, 101 articles were full text screened. Eight studies were included. Patients with Charcot-Marie-Tooth (CMT), chronic inflammatory polyneuropathy (CIDP) and diabetic polyneuropathy (DPN) were represented in the studies (five RCTs, two case-series, and one cross-over trial). The methodological quality ranged from fair-poor in seven studies, one study reached good quality. Results from the studies indicated that strength training in CMT, CIDP and DPN may improve strength. However, various outcomes were used to evaluate strength training, so direct comparisons were difficult.

**Discussion:** In this scoping review we summarized research on strength training and outcomes evaluated in interventions in patients with PNP. Eight studies were included, they indicated that strength training may be beneficial for patients with PNP. However, due to low methodological strength of most studies a recommendation for patients with PNP cannot be made. Thus, the low number of studies with relatively low quality, where various functional outcomes were used, underscores the importance of future studies to evaluate the effect of strength training on relevant functional outcomes and strength in patients with PNP.

## 1 Introduction

Polyneuropathy (PNP) is a disorder that leads to damage of sensory, motor and/or autonomic peripheral nerves over time (Sommer et al., 2018). The prevalence of PNP is estimated to 5.5% in the middle-aged population and increases with age to 13% over the age of 70 (Hanewinckel et al., 2016). However, PNP may be vastly underdiagnosed and studies have suggested that the prevalence may be as high as 9.4% in the middle-aged population (Hanewinckel et al., 2016). PNP can be idiopathic, acquired, or hereditary (Sommer et al., 2018; Lehmann et al., 2020). Depending on the etiology of PNP, progression in symptoms can be anything from rapid (over weeks to months) to slow (over years to decades) before physical impairment occurs (Sommer et al., 2018; Lehmann et al., 2020). Symptoms vary in severity and form, from predominantly sensory symptoms (paresthesia, numbness) in hands or feet to severe sensorimotor affection with both proximal and distal involvement (Hoffman et al., 2015; Sommer et al., 2018; Lehmann et al., 2020). Irrespective of cause, PNP often results in considerable functional impairments, i.e., increased tendency to fall, dependency on walking aids, inability to ascend-descend stairs and difficulties in activities of daily living (Callaghan et al., 2015; Hoffman et al., 2015). Strength training has been shown to improve strength, gait and functional outcomes in healthy young and older adults (de Vos et al., 2005; Liu and Latham, 2009; Garber et al., 2011; Raymond et al., 2013; Borde et al., 2015; Hvid et al., 2016; Chen et al., 2021), patients with stroke (Hill et al., 2012) and patients with multiple sclerosis (Dalgas et al., 2009; Gomez-Illan et al., 2020). Since treatment only exists for very few percentages of the potential underlying causes (inflammatory) it is obvious to consider if strength training could be a potential treatment to counteract functional impairments and loss of physical capacity in patients with PNP. To date very few studies have investigated the effect of strength training in patients with PNP (White et al., 2004; Smith and Mulligan, 2014; Corrado et al., 2016; Fuller et al., 2016) and there is no consensus on the extent to which strength training may improve physical capacity and activities of daily living in patients with PNP. Thus, the aim of this scoping review was to summarize research on strength training interventions and outcomes on physical function for patients with PNP of different etiology. The outcome of this review may help the design of future strength training interventions in patients with PNP.

## 2 Methods

### 2.1 Study design

This scoping review was performed following the Preferred Reporting Items for Systematic reviews and Meta-analysis extension for Scoping reviews (PRISMA-ScR) Checklist (Tricco et al., 2018). The review protocol was not preregistered.

### 2.2 Eligibility criteria

Studies were included if they evaluated a protocol of strength training for patients with immune-mediated, hereditary, metabolic or idiopathic polyneuropathy. To be considered as a strength training intervention there had to be a clearly described strength training protocol (load ≥70% of 1 repetition maximum (RM)), with 1RM defined as the maximum load that can be lifted through the full range of motion one time (American College of Sports Medicine position stand, 2009; Verdijk et al., 2009). Loads ≥70% of 1RM were chosen to follow recommended intensities that are necessary in order to obtain increase in muscle strength and muscle hypertrophy (American College of Sports Medicine position stand, 2009). Studies evaluating outcomes on physical function with both clinician and patient reported outcome measures were considered eligible. Studies published in English, Danish, Swedish, or Norwegian were included. Studies in children and adolescents (<18 years) and in patients with polyneuropathy resulting from critical illness poly-myopathy were excluded. Conference abstracts, case studies based on a single participant, study protocols and expert opinions were also excluded.

### 2.3 Information sources and search

The search strategy was developed by BSP with assistance from TD and an information specialist from Copenhagen University Library. The databases Pubmed, Embase, Cinahl, Cochrane and Web of Science were searched in the period from 16th November 2022 to 25th November 2022. To broaden the search, the PICO (Richardson et al., 1995) was limited to only Population (patients with PNP) and Intervention (strength training), leaving out comparison and outcome since the aim of this study was to summarize research in strength training for patients with PNP, and research within this field is limited. The full search query is available in Supplementary material (Supplementary Tables S1–S4).

Reviews on training and PNP retrieved from the search were read and references checked for studies fulfilling inclusion criteria for this scoping review.

### 2.4 Selection of articles

Three of the authors (BSP, LSK and ABK) used Covidence (Covidence, 2022) to independently screen studies. After each step (title-abstract screening and full-text screening) disagreements were resolved by discussion until agreement or by consulting a fourth reviewer (TD).

### 2.5 Data items

Data were extracted from the included studies by BSP and ABK. Data of interest were information on how strength training was planned (i.e., load (RM), number of sets, progression, supervised or unsupervised, location of training, sessions pr. week and duration) and how the interventions were evaluated (outcome measures and results). Data were summarized and presented in tables sorted by population.

### 2.6 Critical appraisal

Downs and Blacks checklist (Downs and Black, 1998; Deeks et al., 2003) was used to assess the quality of the included studies. The 27 items of the checklist evaluate quality within reporting, external validity, internal validity, confounding and power. We used a modified version of the checklist, where item 27 (on study power) was modified and evaluated with one point (if power was reported) or zero points (if power was not reported or reported unclearly) instead of a score between 0–5 points. The modified version has previously been used (Aubut et al., 2013). The modified total score ranges from 0–28, where highest methodological quality is scored highest. In previous studies, the quality has been considered excellent at a score between 26–28 points, good at a score between 20–25, fair at a score between 15–19 and poor at a score ≤14 points (Hooper et al., 2008; Silverman et al., 2012). Two reviewers (BSP and ABK) evaluated and rated the studies.

## 3 Results

### 3.1 Selection of studies

**FIGURE 1:**
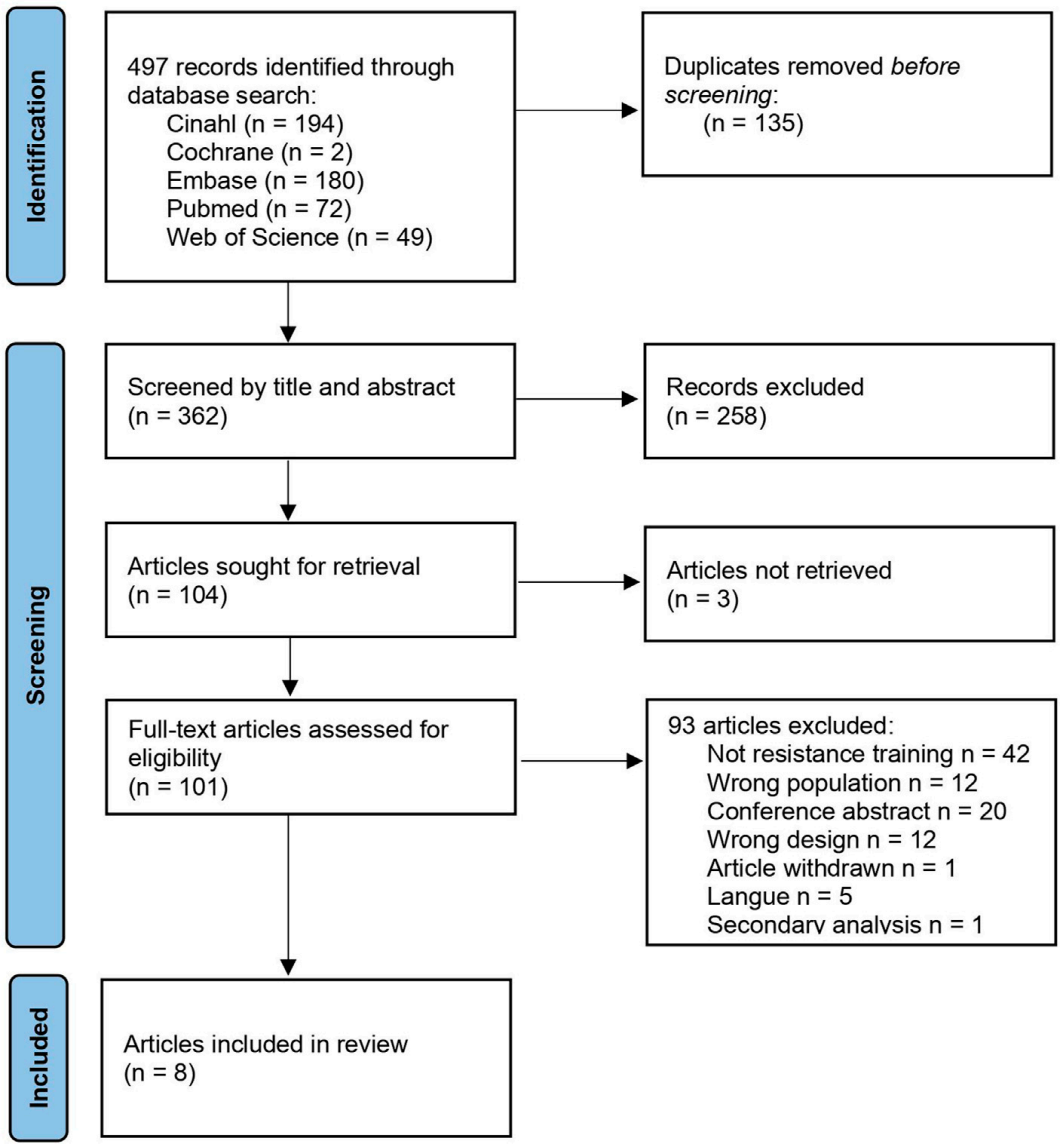
PRISMA-ScR flow diagram of study selection.

497 studies were identified through search in databases (Figure 1). After duplicates were removed, three reviewers screened 362 articles by title and abstract of which 101 articles were retrieved and full text screened by the three reviewers. After full-text screening, eight studies fulfilled the inclusion criteria and were included in this scoping review (Figure 1).

### 3.2 Characteristics of the studies

The eight studies included in this review evaluated strength training in patients with Charcot-Marie-Tooth (CMT) (n = 2 studies), Chronic Inflammatory Demyelinating Polyneuropathy (CIDP) (n = 1 study) and diabetic polyneuropathy (DPN) (n = 5 studies). The designs of the studies were: RCTs (n = 5), case series (n = 2) and cross over trial (n = 1). A total of 209 patients with PNP (CMT n = 34, CIDP n = 18 and DPN n = 157) participated in the eight interventions (Tables 1, 2).

**TABLE 1:** Characteristics of the interventions.

Author	Participants	Intervention (strength training exercises)	Sets and load (RM)	RM test	Supervised training	Adherence reporting	Location	Sessions pr week	Weeks
Hackett et al. (2021)	CMT n = 4	Leg press, leg FLX-EXT, hip ABD, chest press, seated row, ankle PF and DF	Progression from 50% of 1RM to 80% of 1RM	8RM test	Yes	Attendance rate 100%	UNI	3	8
Lindeman et al. (1995)	CMT and MyD n = 66 (n = 30 CMT)	Knee EXT and FLX, hip EXT and ABD	Week1-8: 60% of 1RM	ND 1RM	NO	TD Attendance not reported	HT	3	24
			Week 9-16: 70% of 1RM						
			Week 17-24: 80% of 1RM						
			Load adjustment supervised						
Markvardsen et al. (2018)	CIDP (treated with SCIG) n = 18	Knee EXT and FLX, elbow EXT and FLX. Unilateral exercises for weakest side, untrained side as control	3 sets of 12RM Load adjustment supervised every 1–2 weeks	12RM test	NO	TD Attendance 30 ± 11 sessions	FC	3	12
Handsaker et al. (2016)	Diabetes and DPN n = 43 (n = 9 DPN)	Leg EXT, leg press and ankle press	3 sets of 12RM	12RM test	Yes	ND	ND	1	16
Handsaker et al. (2019)	Diabetes and DPN n = 40 (n = 10 DPN)	Leg EXT, leg press and ankle press	3 sets of 12RM Adjustment of load and techniques the first 2 weeks	12RM test	Yes	ND	UNI	1	16
Khan et al. (2022)	DPN n = 109	Leg press, bench-press, pull-downs, abdominal crunches, knee EXT and FKX, ankle PF and DF, back EXT	Week1-2: 3 sets of 15RM	8RM test	Yes	Attendance median of 29 sessions	UNI	2–3	12
			Week 3-4: 3 sets of 12RM						
			Week 5-8: 3 sets of 10RM						
			Week 9-12: 3 sets of 8 RM						
Lindberg et al. (2020)	DPN and stable foot ulcer n = 5	Knee EXT and FLX, hip ABD, low row	Week1-3: 3 sets of 15RM	8RM test	Yes	Attendance rate 92%	ORC	ND	10
			Week 4-10: 3 sets of 10RM						
Seyedizadeh et al. (2020)	DPN n = 24	Chest press, pull-down, sit-ups, triceps press, barbell-curl, leg EXT, leg curls, push-ups	Week1-4: 2 sets of 12RM	12RM test	ND	ND	ND	3	8
			Week 5-8: 3 sets of 12RM						

CMT: Charcot-Marie-Tooth; MyD: myotonic dystrophy; CIDP: chronic inflammatory demyelinating polyneuropathy; SCIG: subcutaneous immunoglobulin; DPN: diabetic polyneuropathy; FLX: flexion; EXT: extension; ABD: abduction; PF: plantar flexion; DF: dorsi flexion; RM: repetition maximum; NO: unsupervised; TD: training dairy; UNI: training at university; HT: home training; FC: training at fitness center; ORC: outpatient rehabilitation center; ND: not described.

**TABLE 2:** Strength training interventions—outcome measures and results.

Author	Participants	Outcome measure	Results
Hackett et al. (2021) Case-series	CMT n = 4	1RM (knee, hip, chest) Stair climb test Static balance SF-36 FES-I 6-MWT, 2-MWT Tandem walk	Power training was tolerated by n = 4 Improvements in: Leg extension: 35.2% ± 30.0% (n = 3)*, Leg flexion: 8.9% ± 3.3% (n = 4)*, Leg-press: 11.6% ± 18.3% (n = 2)*, Hip abd: (R): 34.1% ± 31.0% (=4)*, (L): 70.0% ± 46.3% (n = 4)* 2MWT: 3.9% ± 3.8% (n = 3)*, FES: −6.7% ± 15.4% (n = 3)*, Static balance: 12.8% ± 12.6% (n = 4)* Tandem walk: −26.9% ± 23.3% (n = 4) SF-36p and 6MWT improved in n = 2, decreased in n = 2
Lindeman et al. (1995) RCT	CMT, MyD n = 66 (n = 30 with CMT)	IKS (knee) Isometric MVC 6MeWT, 50MWT Stair climb test Rising from chair Rising from supine WOMAC	Isokinetic strength (knee EXT) +14% (*p* = 0.01)** Isokinetic strength (knee FLX) +13% (*p* = 0.07)** 6MeWT (*p* = 0.01)*** The remaining outcomes were unchanged
Markvardsen et al. (2018) Cross-over	CIDP (treated with SCIG) n = 18	IKS (knee + elbow) ODSS, SF-36, FSS, 6MWT	IKS (knee FLX, EXT) 17.2% ± 22.8%, *p* = 0.001** IKS (knee + elbow) 13.8% ± 16.0%, *p* < 0.05 6MWT, ODSS, SF-36 and FSS were unchanged
Handsaker et al. (2016) RCT	Diabetes, DPN n = 43 (n = 9 with DPN)	Body movement, ground reaction force and muscle activity	Speed of strength generation (knee and ankle) improved: *p* < 0.01***
Handsaker et al. (2019) RCT	Diabetes, DPN n = 40 (n = 10 with DPN)	Accuracy of foot placement during walking	Accuracy of stepping increased 45%, *p* > 0.05
Khan et al. (2022) RCT	DPN n = 109	ISK, IENFD 6MWT, FTSST Postural stability DN4, SF-12, FES-I MDI, FSS, MNSI-q	ISK (knee) improved: 10.3 Nm ±9.6 Nm, *p* = 0.002# 6MWT improved: 34.6 m ± 40.9 m, 0.001 FTSST improved: −1.5 s ± 4.6 s, *p* = 0.02 No change was found in IENFD, SF-12, FSS, MNSI-q, FES-I or postural stability
Lindberg et al. (2020) Case-series	DPN with stable foot ulcer n = 5	Adherence Foot ulcers size ISM (HHD) Tandem test Astrand bicycle test	Adherence: n = 5 patients completed Attendance rate: 92%, Training was progressed. Safety: for n = 5 patients foot ulcer decreased in size ISM (knee EXT): 20.2 Nm ±19.1Nm No change in Tandem test and Aastrand test
Seyedizadeh et al. (2020) RCT	DPN n = 24	KinesinLC, 6MWT Biceps curls Chair-stand-test	Chair-stand-test improved: *p* = 0.01*** No change in serum kinesinLC, 6MWT or biceps curls

CMT: Charcot-Marie-Tooth; MyD: myotonic dystrophy; CIDP: chronic inflammatory demyelinating polyneuropathy; SCIG: subcutaneous immunoglobulin; DPN: diabetic polyneuropathy; SF-36: Health related quality of life; ODSS: overall disability sum score; FES-I: falls efficacy scale international, 6MWT: 6-Munite Walk Test; 2MWT: 2-m Walk Test; IKS: isokinetic strength; MVC: maximum voluntary contraction; 6MeWT: 6-m Walk Test 50MWT: 50-m Walk Test; WOMAC: WOMAC, questionnaire; IENFD: intra epidermal nerve fiber density; FTSST: Five-Times-Sit-to-Stand-Test; DN4: neuropathic pain; SF-12: Health related quality of life; MDI: major depression inventory; FSS: fatigue severity scale; MNSI-q: Neuropathy symptoms; ISM: isometric strength; HHD: hand held dynamometer; kinesinLC: kinesin light chain; * Improvement of 1RM, mean change in % ±SD; ** Improvement in % and/or (*p*-value); *** only *p*-value reported; # mean difference ±SD (95% CI), *p*-value.

### 3.3 Critical appraisal

The methodological quality assessment of the studies is presented in Tables 3, 4. Of the five RCTs, one article (Khan et al., 2022) scored 23 points (good). For the remaining four RCTs (Lindeman et al., 1995; Handsaker et al., 2016; Handsaker et al., 2019; Seyedizadeh et al., 2020) the score ranged from 9 to 15 points (fair-poor). The two case-series (Lindberg et al., 2020; Hackett et al., 2021) and one cross-over-trial (Markvardsen et al., 2018) were also assessed using Downs and Blacks checklist, with scores ranging from 13 to 18 points (fair-poor).

**TABLE 3:** Sum of quality assessment of randomized controlled trials—Downs and Blacks checklist.

Author	Reporting	External validity	Internal validity	Internal validity confounding	Power	Total score downs and black
Lindeman et al. (1995) RCT	7	1	5	2	0	15
Handsaker et al. (2016) RCT	4	0	4	2	0	10
Handsaker et al. (2019) RCT	4	0	3	2	0	9
Khan et al. (2022) RCT	11	1	6	4	1	23
Seyedizadeh et al. (2020) RCT	5	0	3	1	0	9

**TABLE 4:** Sum of quality assessment of case-series and cross-over trial—Downs and Blacks checklist.

Author	Reporting	External validity	Internal validity	Internal validity confounding	Power	Total score downs and black
Hackett et al. (2021) Case	8	0	3	2	0	13
Markvardsen et al. (2018) Cross-over	10	2	4	2	0	18
Lindberg et al. (2020) Case	8	3	4	3	0	18

### 3.4 Strength training interventions and polyneuropathy

The length of the strength training interventions ranged from eight to 24 weeks (mean length of interventions 13 weeks), with one-three sessions per week. In five studies, strength training was supervised (Handsaker et al., 2016; Handsaker et al., 2019; Lindberg et al., 2020; Hackett et al., 2021; Khan et al., 2022), in two studies patients trained unsupervised but adjustments in load were supervised every other week (Lindeman et al., 1995; Markvardsen et al., 2018), and in one study it was unclear if training was supervised (Seyedizadeh et al., 2020). The locations of the strength training were either the patient’s home (n = 1) (Lindeman et al., 1995), a local fitness center (n = 1) (Markvardsen et al., 2018), an outpatient rehabilitation center (n = 1) (Lindberg et al., 2020), or a university (n = 3) (Handsaker et al., 2019; Hackett et al., 2021; Khan et al., 2022). In two studies, location was unclear (Handsaker et al., 2016; Seyedizadeh et al., 2020). Most of the studies focused on exercises for the lower limb (Lindeman et al., 1995; Handsaker et al., 2016; Markvardsen et al., 2018; Handsaker et al., 2019; Lindberg et al., 2020), but in three studies focus was on exercises for upper and lower limb, back, chest and shoulders (Seyedizadeh et al., 2020; Hackett et al., 2021; Khan et al., 2022). In four studies, weights were gradually progressed to reach higher percentage of 1RM (Lindeman et al., 1995; Lindberg et al., 2020; Hackett et al., 2021; Khan et al., 2022), and in four studies weights were adjusted to maintain the same percentage of 1RM (Handsaker et al., 2016; Markvardsen et al., 2018; Handsaker et al., 2019; Seyedizadeh et al., 2020). In three studies, the 8-repetition maximum test was used to calculate 1RM (Lindberg et al., 2020; Hackett et al., 2021; Khan et al., 2022), in four studies a 12-repetition maximum test was used to calculate 1RM (Handsaker et al., 2016; Markvardsen et al., 2018; Handsaker et al., 2019; Seyedizadeh et al., 2020), and in one study it was unclear how 1RM was calculated (Lindeman et al., 1995). Adherence to training was reported in four of the studies (Markvardsen et al., 2018; Lindberg et al., 2020; Hackett et al., 2021; Khan et al., 2022). In one study adherence to training was registered in training dairies but not reported in the article (Lindeman et al., 1995). In the two studies with unsupervised training, adherence to training but not training intensity was registered in training dairies (Lindeman et al., 1995; Markvardsen et al., 2018), but only one of the studies reported data regarding adherence (Markvardsen et al., 2018). Four studies did not report adherence to training (Lindeman et al., 1995; Handsaker et al., 2016; Handsaker et al., 2019; Seyedizadeh et al., 2020). Below, the strength training interventions from the included studies are sorted and presented by population. For description of the interventions see Tables 1, 2.

### 3.5 Charcot-Marie-Tooth (CMT)

Two studies evaluated strength training for patients with CMT (Lindeman et al., 1995; Hackett et al., 2021). In the RCT by Lindeman et al. (1995), training was primarily focused on improving strength of the lower limb. Training load was progressed during the intervention period from 50% to a maximum of 80% of 1RM. In both studies, strength training was tolerated by patients with CMT. In the RCT by Lindeman et al. (1995), isokinetic knee strength improved (extension 14%, flexion +13%) during the 24 weeks of training (Tables 1, 2). In the case-series by Hackett et al. (2021), three of four patients improved strength of the knee, and two of four patients improved leg-press strength during the 8 weeks of training (Hackett et al., 2021) (Tables 1, 2).

### 3.6 Chronic inflammatory demyelinating polyneuropathy (CIDP)

Strength training for patients with CIDP was evaluated in one study by Markvardsen et al. (2018). Patients with CIDP trained unilateral strength of the knee and elbow with the other side serving as a control. The weights were adjusted throughout the intervention period to maintain a load at three sets of 12RM. Strength of the knee (trained side) improved (17.2% ± 22.8%) after the 12 weeks of strength training (Tables 1, 2).

### 3.7 Diabetic polyneuropathy (DPN)

The effect of strength training in patients with DPN was evaluated in five of the included studies. Two studies (Seyedizadeh et al., 2020; Khan et al., 2022) conducted strength training exercises in both the upper and lower body, whereas three studies (Handsaker et al., 2016; Handsaker et al., 2019; Lindberg et al., 2020) mainly focused on lower body exercise training. In two studies (Lindberg et al., 2020; Khan et al., 2022), the load was gradually progressed by increasing weight to reach a higher percentage of 1RM - weight increased from three sets of 15RM to three sets of 8RM (Khan et al., 2022) and three sets of 10RM (Lindberg et al., 2020). In the remaining three studies (Handsaker et al., 2016; Handsaker et al., 2019; Seyedizadeh et al., 2020), weights were adjusted to maintain load at three sets of 12RM. In the RCT by Khan et al. (Khan et al., 2022), significant improvements were seen in knee strength (+10.3 Nm ±9.6 Nm), 6-min walk test (6MWT) (34.6 m ± 40.9 m) and Five-Times-Sit-To-Stand test (−1.5 s ± 4.6 s) after the 12 weeks strength training intervention (Tables 1, 2). Lindberg et al. (2020) reported that non-weightbearing progressive strength training was safe in the five included patients with DPN with stable foot ulcers. In two studies by Handsaker et al. (2016); (Handsaker et al., 2019), elements of gait and stair climb were evaluated after strength training, and they found that speed of strength generation and accuracy of stepping were increased after 16 weeks of strength training (Tables 1, 2).

### 3.8 Strength training and functional outcomes

Functional outcomes were evaluated in five studies (Lindeman et al., 1995; Markvardsen et al., 2018; Seyedizadeh et al., 2020; Hackett et al., 2021; Khan et al., 2022) (Table 1; Table2) by the following standardized tests: 6-min walk test (n = 4) (Markvardsen et al., 2018; Seyedizadeh et al., 2020; Hackett et al., 2021; Khan et al., 2022), 2-m walk test (n = 1) (Hackett et al., 2021), 6-m walk test (n = 1) (Lindeman et al., 1995), 50-m walk test (n = 1) (Lindeman et al., 1995), five-times-sit-to-stand-test (n = 1) (Khan et al., 2022), and the 30-second-chair-stand-test (n = 1) (Seyedizadeh et al., 2020). Besides from the standardized tests, functional performance was evaluated in the following activities: rising from a chair (n = 1) (Lindeman et al., 1995), rising from supine (n = 1) (Lindeman et al., 1995), and stair climbing (n = 2) (Lindeman et al., 1995; Hackett et al., 2021). None of the eight studies used functional outcomes to evaluate upper limb function including fine motor skills. In studies where functional outcomes were evaluated, improvement was seen in: 6-m walk test (n = 1) (Lindeman et al., 1995), 6-min walk test (n = 1) (Khan et al., 2022), five-times-sit-to-stand (n = 1) (Khan et al., 2022), 30-second-chair-stand-test (n = 1) (Seyedizadeh et al., 2020). No changes were seen in functional performance evaluated in the activities: rising from a chair (n = 1) (Lindeman et al., 1995), rising from supine (n = 1) (Lindeman et al., 1995), stair climb (n = 2) (Lindeman et al., 1995; Hackett et al., 2021), or the 50-m walk test (Lindeman et al., 1995).

### 3.9 Strength training and patient reported outcome measures (PROM)

In four of the eight studies, patient reported outcome measures were evaluated (Lindeman et al., 1995; Markvardsen et al., 2018; Hackett et al., 2021; Khan et al., 2022). The Western Ontario McMaster Universities Arthritis Index (WOMAC) was used to evaluate PROM in one study with patients with Charcot-Marie-Tooth and Myotonic Dystrophy (Lindeman et al., 1995). The Overall Disability Sum Score (ODSS) was used in one study to evaluate disability in patients with chronic inflammatory demyelinating polyneuropathy (Markvardsen et al., 2018). Health related quality of life was evaluated in two studies (Markvardsen et al., 2018; Khan et al., 2022). In the study by Markvardsen et al. (2018), the Short Form-36 (SF-36) was used in patients with chronic inflammatory demyelinating polyneuropathy, and in the study by Khan et al. the shorter version, Short Form-12 (SF-12), was used in patients with diabetic polyneuropathy (Khan et al., 2022). Fear of falling was evaluated in two studies using the Falls Efficay Scale-International (FES-I) (Hackett et al., 2021; Khan et al., 2022). The Fatigue Severity Scale (FSS) was used to evaluate fatigue in two studies, one in patients with chronic inflammatory demyelinating polyneuropathy (Markvardsen et al., 2018) and one in patients with diabetic polyneuropathy (Khan et al., 2022). The Michigan Neuropathy Screening Instrument (MNSI) is a combined PROM and examination tool to assess distal symmetric peripheral neuropathy in patients with diabetes, and was used in one study (Khan et al., 2022). The Douleur Neuropathique en 4 Questions (DN4) was used to evaluate neuropathic pain in one study (Khan et al., 2022). All patient reported outcome measures evaluated in the four studies were unchanged after the strength training interventions (Lindeman et al., 1995; Markvardsen et al., 2018; Hackett et al., 2021; Khan et al., 2022) (Table 1; Table 2).

## 4 Discussion

In this scoping review, the aim was to include research studies investigating strength training and effect on physical function in patients with PNP of different etiology. Of the eight studies included in this review, only one RCT was powered and had methodological quality to detect a difference in and conclude on the primary outcome. This study points toward a beneficial effect on both strength and functional outcomes after 12 weeks of strength training at an intensity of 80% of 1RM. Due to the low methodological quality of most of the studies, the discussion and conclusion below will focus on planning of strength training interventions and choice of outcome measures rather than on the effect of strength training.

### 4.1 Training intensities

In healthy adults, the improvement in outcomes of a training intervention depends on the length of the intervention and to an even greater extent, the training intensity (Garber et al., 2011). Moreover, a review examining the effect of strength training in patients with multiple sclerosis reported that gait speed and endurance only improved in studies where intensities exceeded 70% of 1RM (Mañago et al., 2019; Andreu-Caravaca et al., 2022). Also, a study examining the effect of strength training in stroke survivors only found improvement in strength and gait function when the training intensity exceeded 80% of 1RM (Hill et al., 2012). Thus, it seems that in patients with central neurological disorders, the intensity of training bouts is just as important as in healthy adults. In only three (two RCTs and one case-series) of the eight included studies in this review, patients with PNP reached a training intensity of 80% of 1RM (Lindeman et al., 1995; Hackett et al., 2021; Khan et al., 2022), and in only two of the three studies, training was supervised (Hackett et al., 2021; Khan et al., 2022). In the RCT study by Khan et al. (2022) where the training intensity reached >80% in patients with DPN, significant improvements were seen in strength in the lower limb along with functional improvement in terms of longer and more stable gait function. In the two other studies with a training intensity >80% of 1RM, only the RCT study by Lindeman et al. (1995) found a moderate effect on knee extension strength, but it was not transferred to functional outcomes (Lindeman et al., 1995; Hackett et al., 2021). One explanation for the lack of improvement in functional outcomes and relatively moderate improvement in strength could, at least in the study by Lindeman et al. (1995), relate to the training being unsupervised and thus training intensity solely self-reported. It is well-documented that the effect of unsupervised training in general has poorer outcome than supervised training (Lacroix et al., 2016; Lacroix et al., 2017; Tsekoura et al., 2018), which in this case may have inflicted on the actual training intensity and thus effect of training (Lindeman et al., 1995). In the case-series by Hackett et al. (2021), all training bouts were supervised, but since the design was case-series they could not conclude on the effect of 8 weeks of high intensity strength training, but the authors reported that high intensity training was safe and well tolerated by patients with CMT (Hackett et al., 2021). It is unknown whether all patients with PNP may benefit from strength training or if improvement potential relates to the etiology of PNP. In previous studies on exercise and training in patients with PNP, aerobic training, strength training or a combination of both (Otterman et al., 2011; Kluding et al., 2012; Nadi et al., 2017) have been practiced. In these studies, however, training intensities did not reach levels that are associated with a potential effect on strength in healthy (American College of Sports Medicine position stand, 2009) or patients with other neurological disorders (Hill et al., 2012; Mañago et al., 2019; Andreu-Caravaca et al., 2022). Interestingly, in two studies, strength and functional outcomes were not even investigated after the training period (Kluding et al., 2012; Nadi et al., 2017). The diverse and overall conflicting results from the different training studies underscore that when investigating effect of strength training in patients with PNP, different training protocols including different strength training intensities are warranted to evaluate the optimal dose and the effect on strength and functional outcomes in patients with PNP of different etiology.

### 4.2 Outcome measures

Compared to healthy persons, patients with PNP can have considerable neurological impairments due to muscle weakness and sensory disturbances that can affect both activities of daily living and participation (Hoffman et al., 2015). Among others, they are at increased risk of having impaired gait function, impaired balance, and increased risk of falling (Lindeman et al., 1995; Callaghan et al., 2015; Khan and Andersen, 2022). This is important to take into account when evaluating interventions in PNP. Therefore, in strength training interventions, it could be relevant to use a set of standardized tests that includes evaluation of gait function, balance, and coordination as well as hand function and fine motor skills and not only tests of muscle strength. The 6-min walk test (6MWT), the 10-m walk test (10MWT) and the Six-Spot-Step-Test (SSST) have been shown to be reliable in patients with neuromuscular disorders and other neurological conditions (Watson, 2002; Erdmann et al., 2005; Nieuwenhuis et al., 2006; Erdmann et al., 2017; Knak et al., 2017; Kreutzfeldt et al., 2017; Andersen and Kristensen, 2019; Spina et al., 2019; Vita et al., 2019). The tests evaluate walking capacity, gait speed and coordination and could be included as outcome measures in future strength training interventions. In five of the eight included studies, functional outcome measures were used to evaluate the effect of strength training (Lindeman et al., 1995; Markvardsen et al., 2018; Lindberg et al., 2020; Hackett et al., 2021; Khan et al., 2022). Four different tests were used to evaluate gait function, and transitions and functional lower limb strength were evaluated with four different test procedures while only two of them were standardized tests. In order to evaluate functional impairments and the response to strength training in patients with PNP, it is imperative to use functional outcome measures that are valid, responsive to change, and clinically relevant to both patients and clinicians. Also, it needs to be considered if a given outcome measure is expected to be affected by strength training or the chosen intervention. Thus, it is possible that the lack of effect on functional outcomes seen in the included studies (Lindeman et al., 1995; Seyedizadeh et al., 2020; Hackett et al., 2021) is due to the choice of outcome measure, more than the strength training intervention not being effective. Also, it is possible that strength training in patients with PNP would translate into clinically relevant improvements in functional outcomes with more specific outcome measures. In three studies, upper limb strength was trained without studying a potential relevant functional outcome in the upper limb (Seyedizadeh et al., 2020; Hackett et al., 2021; Khan et al., 2022). The lack of improvement in functional outcomes even in patients with improvement in strength after training underscores the importance of choosing a given outcome that relates to the training intervention.

### 4.3 Patient reported outcome measures (PROMs)

The use of PROMs can add important information on patients’ experience of health status, fatigue, impairments and how these factors affect daily living (Dawson et al., 2010; Basch et al., 2018). When choosing a PROM, it is important that the PROM is relevant and relates to the population and the problem that is being evaluated (Dawson et al., 2010). Moreover, if disease specific the PROM must be validated for that population (Dawson et al., 2010). In four of the included studies, nine different PROMs were used as secondary outcomes. Of those, only two studies used three PROMs that are specific PNP questionnaires (ODSS, DN4 and MNSI-q), whereas three of the studies used five other PROMs (SF-36, FES-I, FSS, SF-12 and MDI) that are generally accepted for all persons despite health status and include health-related questions. Additionally, Lindeman et al. used a PROM that has the purpose to evaluate health status in patients with hip and knee-osteoarthrosis (WOMAC (Gandek, 2015)) to assess health status in patients with PNP (Lindeman et al., 1995). Neither of these studies found an improvement in the PROMs applied. We believe that the lack of improvement in PROMs following strength training in patients with PNP could be caused by the choice of a PROM that could not be expected to change with the intervention. The lack of improvement in PROMs in four out of four studies, emphasizes the importance of choosing the relevant PROMs so that it is both specific for the population and relevant and responsive to the intervention (Churruca et al., 2021).

### 4.4 Strength and limitations

Even though the inclusion criteria for our review were wide, i.e., inclusion of all acquired, hereditary, or idiopathic PNP except critical illness neuro-myopathy and all study designs except single cases, protocols, conference abstracts and expert opinions, only eight studies could be included in this review. The eight studies represented three different study designs (RCT, case-series and cross-over) in patients with PNP of three different subtypes (CMT, CIDP and DPN), and 209 patients with PNP were represented. Thus, a limitation of this review is that there is a very little amount of research within strength training in patients with PNP. Moreover, the representation of patients with PNP in studies investigating the effect of strength training is limited to three subtypes of PNP, and thereby not addressing potential important differences between the effect of strength training in acquired *versus* hereditary PNP. Further, the overall quality of most of the included studies was low (Table 3; Table 4) ranging from 9 to 23 on Downs and Blacks checklist. Only one RCT had good methodological quality. Finally, the included studies used different outcome measures to assess the effect of strength training, which makes it difficult to compare effect across studies. Also, only three of the outcome measures have been shown to be reliable in patients with neuromuscular disorders and other neurological conditions (Watson, 2002; Erdmann et al., 2005; Nieuwenhuis et al., 2006; Erdmann et al., 2017; Knak et al., 2017; Kreutzfeldt et al., 2017; Andersen and Kristensen, 2019; Spina et al., 2019; Vita et al., 2019). Thus, it is possible that an effect of training can be overlooked due an inappropriate choice of outcome measure. The strengths of this review are the broad search strategy, i.e., the systematic search in five databases, and the systematic selection of articles that was performed by three of the authors. Furthermore, the use of Covidence has ensured that the quality of the study selection and appraisal has been checked. This review calls attention to strength training in patients with PNP. We chose to focus on interventions that included strength training. The reason for this approach was to ensure that the effect of training could be directly compared between studies and related to the primary and secondary outcomes. Therefore, we believe that the choice of addressing the effect of strength training in patients with PNP alone is a strength of this review, rather than a limitation.

## 5 Conclusion

In this scoping review, we summarized research on strength training interventions and outcomes on physical function in patients with PNP of different etiology. In total, eight studies were included, and overall, they indicate that strength training may be beneficial in patients with PNP on strength and functional outcomes. However, only one study was powered and had methodological quality to detect differences in strength and functional outcomes, indicating that 12 weeks of strength training improves strength and gait function in patients with DPN. To evaluate and compare future strength training interventions in patients with PNP, it is relevant to consider patient specific training interventions and outcome measures.

The eight studies represent only three subtypes of PNP, and the strength of the study designs was low. This underscores the importance of future studies on strength training in patients with PNP to investigate the effect on strength, but also on clinically relevant functional outcomes and PROMs in a broad spectrum of causes.

## Data Availability

The original contributions presented in the study are included in the article/Supplementary Material, further inquiries can be directed to the corresponding author.

## References

[B1] American College of Sports Medicine position stand (2009). Progression models in resistance training for healthy adults. Med. Sci. Sports Exer 41 (3), 687–708. 10.1249/MSS.0b013e3181915670 19204579

[B2] Andersen C. W. Kristensen M. T. (2019). Performance stability and interrater reliability of culturally adapted 10-meter walking test for Danes with neurological disorders. J. Stroke Cerebrovasc. Dis. Sept. 28 (9), 2459–2467. 10.1016/j.jstrokecerebrovasdis.2019.06.021 31281111

[B3] Andreu-Caravaca L. Ramos-Campo D. J. Chung L. H. Martínez-Rodríguez A. Rubio-Arias J. Á. (2022). Effects and optimal dosage of resistance training on strength, functional capacity, balance, general health perception, and fatigue in people with multiple sclerosis: A systematic review and meta-analysis. Disabil. Rehabil. 2022, 1–13. 10.1080/09638288.2022.2069295 35579532

[B4] Aubut J. A. L. Marshall S. Bayley M. Teasell R. W. (2013). A comparison of the PEDro and Downs and Black quality assessment tools using the acquired brain injury intervention literature. NeuroRehabilitation 32 (1), 95–102. 10.3233/NRE-130826 23422462

[B5] Basch E. Barbera L. Kerrigan C. L. Velikova G. (2018). Implementation of patient-reported outcomes in routine medical care. Am. Soc. Clin. Oncol. Educ. Book 38, 122–134. 10.1200/EDBK_200383 30231381

[B6] Borde R. Hortobágyi T. Granacher U. (2015). Dose-response relationships of resistance training in healthy old adults: A systematic review and meta-analysis. Sports Med. 45 (12), 1693–1720. 10.1007/s40279-015-0385-9 26420238 PMC4656698

[B7] Callaghan B. Kerber K. Langa K. M. Banerjee M. Rodgers A. McCammon R. (2015). Longitudinal patient-oriented outcomes in neuropathy: Importance of early detection and falls. Neurology 85 (1), 71–79. 10.1212/WNL.0000000000001714 26019191 PMC4501944

[B8] Chen N. He X. Feng Y. Ainsworth B. E. Liu Y. (2021). Effects of resistance training in healthy older people with sarcopenia: A systematic review and meta-analysis of randomized controlled trials. Eur. Rev. Aging Phys. Act. 18 (1), 23. 10.1186/s11556-021-00277-7 34763651 PMC8588688

[B9] Churruca K. Pomare C. Ellis L. A. Long J. C. Henderson S. B. Murphy L. E. D. (2021). Patient-reported outcome measures (PROMs): A review of generic and condition-specific measures and a discussion of trends and issues. Health Expect. 24 (4), 1015–1024. 10.1111/hex.13254 33949755 PMC8369118

[B10] Corrado B. Ciardi G. Bargigli C. (2016). Rehabilitation management of the charcot-marie-tooth syndrome: A systematic review of the literature. Med. Baltim. 95 (17), e3278. 10.1097/MD.0000000000003278 PMC4998680 27124017

[B11] Covidence (2022). Covidence - better systematic review management. Available at: https://www.covidence.org/ .

[B12] Dalgas U. Stenager E. Jakobsen J. Petersen T. Hansen H. J. Knudsen C. (2009). Resistance training improves muscle strength and functional capacity in multiple sclerosis. Neurology 73 (18), 1478–1484. 10.1212/WNL.0b013e3181bf98b4 19884575

[B13] Dawson J. Doll H. Fitzpatrick R. Jenkinson C. Carr A. J. (2010). The routine use of patient reported outcome measures in healthcare settings. BMJ 340, c186. 10.1136/bmj.c186 20083546

[B14] de Vos N. J. Singh N. A. Ross D. A. Stavrinos T. M. Orr R. Fiatarone Singh M. A. (2005). Optimal load for increasing muscle power during explosive resistance training in older adults. J. Gerontol. A Biol. Sci. Med. Sci. 60 (5), 638–647. 10.1093/gerona/60.5.638 15972618

[B15] Deeks J. J. Dinnes J. D’Amico R. Sowden A. J. Sakarovitch C. Song F. (2003). Evaluating non-randomised intervention studies. Health Technol. Assess. 7 (27), 1–173. 10.3310/hta7270 14499048

[B16] Downs S. H. Black N. (1998). The feasibility of creating a checklist for the assessment of the methodological quality both of randomised and non-randomised studies of health care interventions. J. Epidemiol. Community Health 52 (6), 377–384. 10.1136/jech.52.6.377 9764259 PMC1756728

[B17] Erdmann P. G. Teunissen L. L. van den Berg L. H. Notermans N. C. Schröder C. D. Bongers B. C. (2017). Validity of the shuttle walk test as a functional assessment of walking ability in individuals with polyneuropathy. Disabil. Rehabil. 39 (20), 2112–2118. 10.1080/09638288.2016.1217083 27599252

[B18] Erdmann P. G. van Meeteren N. L. U. Kalmijn S. Wokke J. H. J. Helders P. J. M. van den Berg L. H. (2005). Functional health status of patients with chronic inflammatory neuropathies. J. Peripher Nerv. Syst. 10 (2), 181–189. 10.1111/j.1085-9489.2005.0010208.x 15958129

[B19] Fuller A. A. Singleton J. R. Smith A. G. Marcus R. L. (2016). Exercise in type 2 diabetic peripheral neuropathy. Curr. Geri Rep. 5(3), 150–159. 10.1007/s13670-016-0177-6

[B20] Gandek B. (2015). Measurement properties of the western Ontario and McMaster Universities osteoarthritis Index: A systematic review. Arthritis Care Res. Hob. 67 (2), 216–229. 10.1002/acr.22415 25048451

[B21] Garber C. E. Blissmer B. Deschenes M. R. Franklin B. A. Lamonte M. J. Lee I. M. (2011). American College of Sports medicine position stand, quantity and quality of exercise for developing and maintaining cardiorespiratory, musculoskeletal, and neuromotor fitness in apparently healthy adults: Guidance for prescribing exercise. Med. Sci. Sports Exerc. 43 (7), 1334–1359. 10.1249/MSS.0b013e318213fefb 21694556

[B22] Gomez-Illan R. Reina R. Barbado D. Sabido R. Moreno-Navarro P. Roldan A. (2020). Effects of maximal strength training on perceived-fatigue and functional mobility in persons with relapsing-remitting multiple sclerosis. Med. Kaunas. 56 (12), 718. 10.3390/medicina56120718 PMC7765935 33419374

[B23] Hackett D. Roberts-Clarke D. Halaki M. Burns J. Singh M. F. Fornusek C. (2021). High intensity power training in middle-aged women with charcot–marie–tooth disease: A case series. Int. J. Ther. Rehabilitation 28 (6), 1–12. 10.12968/ijtr.2020.0104

[B24] Handsaker J. C. Brown S. J. Bowling F. L. Maganaris C. N. Boulton A. J. M. Reeves N. D. (2016). Resistance exercise training increases lower limb speed of strength generation during stair ascent and descent in people with diabetic peripheral neuropathy. Diabet. Med. 33 (1), 97–104. 10.1111/dme.12841 26108438

[B25] Handsaker J. C. Brown S. J. Petrovic M. Bowling F. L. Rajbhandari S. Marple-Horvat D. E. (2019). Combined exercise and visual gaze training improves stepping accuracy in people with diabetic peripheral neuropathy. J. Diabetes Complicat. 33 (10), 107404. 10.1016/j.jdiacomp.2019.07.001 31371130

[B26] Hanewinckel R. Drenthen J. van Oijen M. Hofman A. van Doorn P. A. Ikram M. A. (2016). Prevalence of polyneuropathy in the general middle-aged and elderly population. Neurology 87 (18), 1892–1898. 10.1212/WNL.0000000000003293 27683845

[B27] Hill T. R. Gjellesvik T. I. Moen P. M. R. Tørhaug T. Fimland M. S. Helgerud J. (2012). Maximal strength training enhances strength and functional performance in chronic stroke survivors. Am. J. Phys. Med. Rehabil. 91 (5), 393–400. 10.1097/PHM.0b013e31824ad5b8 22357133

[B28] Hoffman E. M. Staff N. P. Robb J. M. St Sauver J. L. Dyck P. J. Klein C. J. (2015). Impairments and comorbidities of polyneuropathy revealed by population-based analyses. Neurology 84 (16), 1644–1651. 10.1212/WNL.0000000000001492 25832668 PMC4409579

[B29] Hooper P. Jutai J. W. Strong G. Russell-Minda E. (2008). Age-related macular degeneration and low-vision rehabilitation: A systematic review. Can. J. Ophthalmol. 43 (2), 180–187. 10.3129/i08-001 18347620

[B30] Hvid L. G. Strotmeyer E. S. Skjødt M. Magnussen L. V. Andersen M. Caserotti P. (2016). Voluntary muscle activation improves with power training and is associated with changes in gait speed in mobility-limited older adults - a randomized controlled trial. Exp. Gerontol. 80, 51–56. 10.1016/j.exger.2016.03.018 27090485

[B31] Khan K. S. Andersen H. (2022). The impact of diabetic neuropathy on activities of daily living, postural balance and risk of falls - a systematic review. J. Diabetes Sci. Technol. 16 (2), 289–294. 10.1177/1932296821997921 33719603 PMC8861804

[B32] Khan K. S. Overgaard K. Tankisi H. Karlsson P. Devantier L. Gregersen S. (2022). Effects of progressive resistance training in individuals with type 2 diabetic polyneuropathy: A randomised assessor-blinded controlled trial. Diabetologia 65 (4), 620–631. 10.1007/s00125-021-05646-6 35048156

[B33] Kluding P. M. Pasnoor M. Singh R. Jernigan S. Farmer K. Rucker J. (2012). The effect of exercise on neuropathic symptoms, nerve function, and cutaneous innervation in people with diabetic peripheral neuropathy. J. Diabetes Complicat. 26 (5), 424–429. 10.1016/j.jdiacomp.2012.05.007 PMC3436981 22717465

[B34] Knak K. L. Andersen L. K. Witting N. Vissing J. (2017). Reliability of the 2- and 6-minute walk tests in neuromuscular diseases. J. Rehabil. Med. 49 (4), 362–366. 10.2340/16501977-2222 28352938

[B35] Kreutzfeldt M. Jensen H. B. Ravnborg M. Markvardsen L. H. Andersen H. Sindrup S. H. (2017). The six-spot-step test - a new method for monitoring walking ability in patients with chronic inflammatory polyneuropathy. J. Peripher Nerv. Syst. 22 (2), 131–138. 10.1111/jns.12210 28407329

[B36] Lacroix A. Hortobágyi T. Beurskens R. Granacher U. (2017). Effects of supervised vs. Unsupervised training programs on balance and muscle strength in older adults: A systematic review and meta-analysis. Sports Med. 47 (11), 2341–2361. 10.1007/s40279-017-0747-6 28573401

[B37] Lacroix A. Kressig R. W. Muehlbauer T. Gschwind Y. J. Pfenninger B. Bruegger O. (2016). Effects of a supervised versus an unsupervised combined balance and strength training program on balance and muscle power in healthy older adults: A randomized controlled trial. Gerontology 62 (3), 275–288. 10.1159/000442087 26645282

[B38] Lehmann H. C. Wunderlich G. Fink G. R. Sommer C. (2020). Diagnosis of peripheral neuropathy. Neurol. Res. Pract. 2, 20. 10.1186/s42466-020-00064-2 33324924 PMC7650053

[B39] Lindberg K. Møller B. S. Kirketerp-Møller K. Kristensen M. T. (2020). An exercise program for people with severe peripheral neuropathy and diabetic foot ulcers - a case series on feasibility and safety. Disabil. Rehabil. 42 (2), 183–189. 10.1080/09638288.2018.1494212 30293458

[B40] Lindeman E. Leffers P. Spaans F. Drukker J. Reulen J. Kerckhoffs M. (1995). Strength training in patients with myotonic dystrophy and hereditary motor and sensory neuropathy: A randomized clinical trial. Arch. Phys. Med. Rehabil. 76 (7), 612–620. 10.1016/s0003-9993(95)80629-6 7605179

[B41] Liu C. J. Latham N. K. (2009). Progressive resistance strength training for improving physical function in older adults. Cochrane Database Syst. Rev. 2009 (3), CD002759. 10.1002/14651858.CD002759.pub2 19588334 PMC4324332

[B42] Mañago M. M. Glick S. Hebert J. R. Coote S. Schenkman M. (2019). Strength training to improve gait in people with multiple sclerosis: A critical review of exercise parameters and intervention approaches. Int. J. MS Care 21 (2), 47–56. 10.7224/1537-2073.2017-079 31049034 PMC6489435

[B43] Markvardsen L. H. Overgaard K. Heje K. Sindrup S. H. Christiansen I. Vissing J. (2018). Resistance training and aerobic training improve muscle strength and aerobic capacity in chronic inflammatory demyelinating polyneuropathy. Muscle Nerve 57 (1), 70–76. 10.1002/mus.25652 28345260

[B44] Nadi M. Marandi S. M. Esfarjani F. Saleki M. Mohammadi M. (2017). The comparison between effects of 12 weeks combined training and vitamin D supplement on improvement of sensory-motor neuropathy in type 2 diabetic women. Adv. Biomed. Res. 6, 55. 10.4103/2277-9175.205528 28553628 PMC5434674

[B45] Nieuwenhuis M. M. Van Tongeren H. Sørensen P. S. Ravnborg M. (2006). The six spot step test: A new measurement for walking ability in multiple sclerosis. Mult. Scler. 12 (4), 495–500. 10.1191/1352458506ms1293oa 16900764

[B46] Otterman N. M. van Schie C. H. M. van der Schaaf M. van Bon A. C. Busch-Westbroek T. E. Nollet F. (2011). An exercise programme for patients with diabetic complications: A study on feasibility and preliminary effectiveness. Diabet. Med. 28 (2), 212–217. 10.1111/j.1464-5491.2010.03128.x 21219432

[B47] Raymond M. J. Bramley-Tzerefos R. E. Jeffs K. J. Winter A. Holland A. E. (2013). Systematic review of high-intensity progressive resistance strength training of the lower limb compared with other intensities of strength training in older adults. Arch. Phys. Med. Rehabil. 94 (8), 1458–1472. 10.1016/j.apmr.2013.02.022 23473702

[B48] Richardson W. S. Wilson M. C. Nishikawa J. Hayward R. S. (1995). The well-built clinical question: A key to evidence-based decisions. ACP J. Club 123 (3), A12–A13. 10.7326/ACPJC-1995-123-3-A12 7582737

[B49] Seyedizadeh S. H. Cheragh-Birjandi S. Hamedi Nia M. R. (2020). The effects of combined exercise training (Resistance-Aerobic) on serum kinesin and physical function in type 2 diabetes patients with diabetic peripheral neuropathy (randomized controlled trials). J. Diabetes Res. 2020, 6978128. 10.1155/2020/6978128 32215272 PMC7085367

[B50] Silverman S. R. Schertz L. A. Yuen H. K. Lowman J. D. Bickel C. S. (2012). Systematic review of the methodological quality and outcome measures utilized in exercise interventions for adults with spinal cord injury. Spinal Cord. 50 (10), 718–727. 10.1038/sc.2012.78 22777488

[B51] Smith M. B. Mulligan N. (2014). Peripheral neuropathies and exercise. Geriatr. Rehabil. 30 (2), 131–147. 10.1097/tgr.0000000000000013

[B52] Sommer C. Geber C. Young P. Forst R. Birklein F. Schoser B. (2018). Polyneuropathies. Dtsch. Arztebl Int. 115 (6), 83–90. 10.3238/arztebl.2018.083 29478436 PMC5832891

[B53] Spina E. Topa A. Iodice R. Tozza S. Ruggiero L. Dubbioso R. (2019). Six-minute walk test is reliable and sensitive in detecting response to therapy in CIDP. J. Neurol. 266 (4), 860–865. 10.1007/s00415-019-09207-1 30721354

[B54] Tricco A. C. Lillie E. Zarin W. O’Brien K. K. Colquhoun H. Levac D. (2018). PRISMA extension for scoping reviews (PRISMA-ScR): Checklist and explanation. Ann. Intern Med. 169 (7), 467–473. 10.7326/M18-0850 30178033

[B55] Tsekoura M. Billis E. Tsepis E. Dimitriadis Z. Matzaroglou C. Tyllianakis M. (2018). The effects of group and home-based exercise programs in elderly with sarcopenia: A randomized controlled trial. J. Clin. Med. 7 (12), 480. 10.3390/jcm7120480 30486262 PMC6306785

[B56] Verdijk L. B. van Loon L. Meijer K. Savelberg H. H. C. M. (2009). One-repetition maximum strength test represents a valid means to assess leg strength *in vivo* in humans. J. Sports Sci. 27 (1), 59–68. 10.1080/02640410802428089 19031334

[B57] Vita G. L. Stancanelli C. Gentile L. Barcellona C. Russo M. Di Bella G. (2019). 6MWT performance correlates with peripheral neuropathy but not with cardiac involvement in patients with hereditary transthyretin amyloidosis (hATTR). Neuromuscul. Disord. marts 29 (3), 213–220. 10.1016/j.nmd.2018.11.002 30718023

[B58] Watson M. (2002). Refining the ten-metre walking test for use with neurologically impaired people - ScienceDirect. Physiotherapy 88, 386–397. 10.1016/S0031-9406(05)61264-3

[B59] White C. M. Pritchard J. Turner-Stokes L. (2004). Exercise for people with peripheral neuropathy. Cochrane Database Syst. Rev. 6 (4), CD003904. 10.1002/14651858.CD003904.pub2 PMC12767756 15495069

