# Supplementary material for: Effect of strength training on functional outcomes and strength in patients with polyneuropathy: A scoping review
Source: Front Physiol. 2023 Apr 6;14:1158039. doi: 10.3389/fphys.2023.1158039 (PMC10116572; doi:10.3389/fphys.2023.1158039)
Supplement: Supplementary file 1 [file Table1.DOCX]

Supplementary Material

Effect of strength training on functional outcomes and strength in patients with polyneuropathy: A scoping review

Britt Stævnsbo Pedersen^1*^, Louise Sloth Kodal^1^, Anna Bundgaard Kaalund^1^, Sonja Holm-Yildiz^1^, Mette Merete Pedersen^2,3^, Tina Dysgaard^1,3^

^1^ Copenhagen Neuromuscular Center, Department of Neurology, Rigshospitalet, Copenhagen University Hospital, Copenhagen, Denmark

^2^Department of Clinical Research and Physical Medicine and Rehabilitation Research Copenhagen (PMR-C), Copenhagen University Hospital, Amager and Hvidovre, Denmark

^3^Department of Clinical Medicine, Faculty of Health and Medical Sciences, University of Copenhagen, Copenhagen, Denmark

*** Correspondence:**Corresponding Author: Britt Stævnsbo Pedersen

Copenhagen Neuromuscular Center, Section 8077, Department of Neurology, Rigshospitalet, Inge Lehmans Vej 8, DK-2100 Copenhagen, Denmark. Phone +45 35458401. Mail: [britt.staevnsbo.pedersen.01@regionh.dk](mailto:britt.staevnsbo.pedersen.01@regionh.dk)

**Supplementary Table 1.** Pubmed search

| **Date** | **Subject** | **Pubmed search** | **Hits** |
| --- | --- | --- | --- |
| 25-11-22 | Polyneuropathy and sub-diagnoses Textword and MeSH terms | "polyneuropath*"[Text Word] OR "cidp"[Text Word] OR "chronic inflammatory demyelinating polyneuropath*"[Text Word] OR "chronic inflammatory demyelinating polyradiculoneuropath*"[Text Word] OR "Guillain-Barre Syndrome"[Text Word] OR "guillain barre neuropath*"[Text Word] OR "aidp"[Text Word] OR "acute inflammatory demyelinating polyneuropath*"[Text Word] OR "multifocal motor neuropath*"[Text Word] OR "vasculitis neuropath*"[Text Word] OR "vasculitic neuropath*"[Text Word] OR "vasculitic polyneuropath*"[Text Word] OR "Non-systemic-vasculitic-neuropathy"[Text Word] OR "diabetic neuropath*"[Text Word] OR "diabetic polyneuropath*"[Text Word] OR "diabetes neuropath*"[Text Word] OR "diabetes polyneuropath*"[Text Word] OR "amyloidosis neuropath*"[Text Word] OR "Charcot Marie Tooth"[Text Word] OR "charcot marie tooth neuropath*"[Text Word] OR "hereditary neuropath*"[Text Word] OR "poems"[Text Word] OR "poems neuropath*"[Text Word] OR "monoclonal gammopath*"[Text Word] OR "Polyneuropathies"[MeSH Terms] OR "Diabetic Neuropathies"[MeSH Terms] OR "amyloid neuropathies, familial"[MeSH Terms] OR "Guillain-Barre Syndrome"[MeSH Terms] OR "Paraneoplastic Polyneuropathy"[MeSH Terms] OR "Amyloid Neuropathies"[MeSH Terms] OR "POEMS Syndrome"[MeSH Terms] OR "polyradiculoneuropathy, chronic inflammatory demyelinating"[MeSH Terms] | 80996 |
| 25-11-22 | Strength training, Textword, Ti/ab and MeSH terms | "Resistance Training"[Text Word] OR "Strength training"[Text Word] OR "Strengthening exercise"[Text Word] OR "Resistance Training"[MeSH Terms] OR "Plyometric Exercise"[MeSH Terms] OR "Blood Flow Restriction Therapy"[MeSH Terms] OR "High-Intensity Interval Training"[MeSH Terms] OR "Endurance Training"[MeSH Terms] | 23823 |
| 25-11-22 | Polyneuropathy and strength training | ("polyneuropath*"[Text Word] OR "cidp"[Text Word] OR "chronic inflammatory demyelinating polyneuropath*"[Text Word] OR "chronic inflammatory demyelinating polyradiculoneuropath*"[Text Word] OR "Guillain-Barre Syndrome"[Text Word] OR "guillain barre neuropath*"[Text Word] OR "aidp"[Text Word] OR "acute inflammatory demyelinating polyneuropath*"[Text Word] OR "multifocal motor neuropath*"[Text Word] OR "vasculitis neuropath*"[Text Word] OR "vasculitic neuropath*"[Text Word] OR "vasculitic polyneuropath*"[Text Word] OR "Non-systemic-vasculitic-neuropathy"[Text Word] OR "diabetic neuropath*"[Text Word] OR "diabetic polyneuropath*"[Text Word] OR "diabetes neuropath*"[Text Word] OR "diabetes polyneuropath*"[Text Word] OR "amyloidosis neuropath*"[Text Word] OR "Charcot Marie Tooth"[Text Word] OR "charcot marie tooth neuropath*"[Text Word] OR "hereditary neuropath*"[Text Word] OR "poems"[Text Word] OR "poems neuropath*"[Text Word] OR "monoclonal gammopath*"[Text Word] OR "Polyneuropathies"[MeSH Terms] OR "Diabetic Neuropathies"[MeSH Terms] OR "amyloid neuropathies, familial"[MeSH Terms] OR "Guillain-Barre Syndrome"[MeSH Terms] OR "Paraneoplastic Polyneuropathy"[MeSH Terms] OR "Amyloid Neuropathies"[MeSH Terms] OR "POEMS Syndrome"[MeSH Terms] OR "polyradiculoneuropathy, chronic inflammatory demyelinating"[MeSH Terms]) AND ("Resistance Training"[Text Word] OR "Strength training"[Text Word] OR "Strengthening exercise"[Text Word] OR "Resistance Training"[MeSH Terms] OR "Plyometric Exercise"[MeSH Terms] OR "Blood Flow Restriction Therapy"[MeSH Terms] OR "High-Intensity Interval Training"[MeSH Terms] OR "Endurance Training"[MeSH Terms]) | 72 |

**Supplementary Table 2.** Web of science search

| **Number** | **Web of science search** | **Hits** |
| --- | --- | --- |
| 1 | ALL=(Polyneuropath*) | 22045 |
| 2 | ALL=(CIDP) | 3785 |
| 3 | ALL=("Chronic inflammatory demyelinating Polyneuropath*") | 2303 |
| 4 | ALL=(“Chronic inflammatory demyelinating polyradiculoneuropath*”) | 1196 |
| 5 | ALL=("Guillain-Barre Syndrome") | 13971 |
| 6 | ALL=("guillain barre neuropath*") | 3 |
| 7 | ALL=(“Multifocal Motor Neuropath*”) | 1479 |
| 8 | ALL=(“Vasculitis neuropath*”) | 30 |
| 9 | ALL=(“Vasculitic neuropath*”) | 525 |
| 10 | ALL=(“Vasculitic polyneuropath*”) | 16 |
| 11 | ALL=(“Non-systemic-vasculitic-neuropathy”) | 35 |
| 12 | ALL=(“Diabetic neuropath*”) | 12865 |
| 13 | ALL=(“Diabetic polyneuropath*”) | 1789 |
| 14 | ALL=(“Diabetes neuropath*”) | 131 |
| 15 | ALL=(“Diabetes polyneuropath*”) | 18 |
| 16 | ALL=(“Amyloidosis neuropath*”) | 10 |
| 17 | ALL=(“Charcot Marie Tooth” ) | 6360 |
| 18 | ALL=(“Hereditary neuropath*”) | 2011 |
| 19 | ALL=(POEMS) | 61354 |
| 20 | ALL=(“POEMS neuropath*”) | 11 |
| 21 | ALL=("monoclonal gammopathy") | 7994 |
| 22 | ALL=("Amyloid Neuropathies") | 65 |
| 23 | ALL=("Paraneoplastic Polyneuropathy") | 32 |
| 24 | #23 OR #22 OR #21 OR #20 OR #19 OR #18 OR #17 OR #16 OR #15 OR #14 OR #13 OR #12 OR #11 OR #10 OR #9 OR #8 OR #7 OR #6 OR #5 OR #4 OR #3 OR #2 OR #1 | 121331 |
| 25 | ALL=("Strength training" ) | 8965 |
| 26 | ALL=("Resistance Training") | 14169 |
| 27 | ALL=("Strengthening exercise") | 683 |
| 28 | #25 OR #26 OR #27 | 21620 |
| 29 | #24 AND #28 | 49 |

**Supplementary Table 3.** Embase search

| **#** | **Embase search** | **Hits** |
| --- | --- | --- |
| 1 | Polyneuropath*.mp. [mp=title, abstract, heading word, drug trade name, original title, device manufacturer, drug manufacturer, device trade name, keyword heading word, floating subheading word, candidate term word] | 36,024 |
| 2 | CIDP.mp. [mp=title, abstract, heading word, drug trade name, original title, device manufacturer, drug manufacturer, device trade name, keyword heading word, floating subheading word, candidate term word] | 4,583 |
| 3 | "Chronic inflammatory demyelinating polyradiculoneuropath*".mp. [mp=title, abstract, heading word, drug trade name, original title, device manufacturer, drug manufacturer, device trade name, keyword heading word, floating subheading word, candidate term word] | 1,886 |
| 4 | "Chronic inflammatory demyelinating polyneuropath*".mp. [mp=title, abstract, heading word, drug trade name, original title, device manufacturer, drug manufacturer, device trade name, keyword heading word, floating subheading word, candidate term word] | 5,423 |
| 5 | "Guillain-Barre Syndrome".mp. [mp=title, abstract, heading word, drug trade name, original title, device manufacturer, drug manufacturer, device trade name, keyword heading word, floating subheading word, candidate term word] | 21,859 |
| 6 | "guillain barre neuropath*".mp. [mp=title, abstract, heading word, drug trade name, original title, device manufacturer, drug manufacturer, device trade name, keyword heading word, floating subheading word, candidate term word] | 6 |
| 7 | aidp.mp. [mp=title, abstract, heading word, drug trade name, original title, device manufacturer, drug manufacturer, device trade name, keyword heading word, floating subheading word, candidate term word] | 986 |
| 8 | "acute inflammatory demyelinating polyneuropath*".mp. [mp=title, abstract, heading word, drug trade name, original title, device manufacturer, drug manufacturer, device trade name, keyword heading word, floating subheading word, candidate term word] | 1,336 |
| 9 | "Multifocal Motor Neuropath*".mp. [mp=title, abstract, heading word, drug trade name, original title, device manufacturer, drug manufacturer, device trade name, keyword heading word, floating subheading word, candidate term word] | 1,514 |
| 10 | "Vasculitis neuropath*".mp. [mp=title, abstract, heading word, drug trade name, original title, device manufacturer, drug manufacturer, device trade name, keyword heading word, floating subheading word, candidate term word] | 56 |
| 11 | "Vasculitic neuropath*".mp. [mp=title, abstract, heading word, drug trade name, original title, device manufacturer, drug manufacturer, device trade name, keyword heading word, floating subheading word, candidate term word] | 647 |
| 12 | "Vasculitic polyneuropath*".mp. [mp=title, abstract, heading word, drug trade name, original title, device manufacturer, drug manufacturer, device trade name, keyword heading word, floating subheading word, candidate term word] | 25 |
| 13 | "Non-systemic-vasculitic-neuropath*".mp. [mp=title, abstract, heading word, drug trade name, original title, device manufacturer, drug manufacturer, device trade name, keyword heading word, floating subheading word, candidate term word] | 66 |
| 14 | "Diabetic neuropath*".mp. [mp=title, abstract, heading word, drug trade name, original title, device manufacturer, drug manufacturer, device trade name, keyword heading word, floating subheading word, candidate term word] | 30,925 |
| 15 | "Diabetic polyneuropath*".mp. [mp=title, abstract, heading word, drug trade name, original title, device manufacturer, drug manufacturer, device trade name, keyword heading word, floating subheading word, candidate term word] | 2,250 |
| 16 | "Diabetes neuropath*".mp. [mp=title, abstract, heading word, drug trade name, original title, device manufacturer, drug manufacturer, device trade name, keyword heading word, floating subheading word, candidate term word] | 207 |
| 17 | "Diabetes polyneuropath*".mp. [mp=title, abstract, heading word, drug trade name, original title, device manufacturer, drug manufacturer, device trade name, keyword heading word, floating subheading word, candidate term word] | 31 |
| 18 | "Amyloidosis neuropath*".mp. [mp=title, abstract, heading word, drug trade name, original title, device manufacturer, drug manufacturer, device trade name, keyword heading word, floating subheading word, candidate term word] | 18 |
| 19 | "Charcot Marie Tooth".mp. [mp=title, abstract, heading word, drug trade name, original title, device manufacturer, drug manufacturer, device trade name, keyword heading word, floating subheading word, candidate term word] | 7,938 |
| 20 | "Charcot Marie Tooth neuropath*".mp. [mp=title, abstract, heading word, drug trade name, original title, device manufacturer, drug manufacturer, device trade name, keyword heading word, floating subheading word, candidate term word] | 678 |
| 21 | "Hereditary neuropath*".mp. [mp=title, abstract, heading word, drug trade name, original title, device manufacturer, drug manufacturer, device trade name, keyword heading word, floating subheading word, candidate term word] | 2,003 |
| 22 | POEMS.mp. [mp=title, abstract, heading word, drug trade name, original title, device manufacturer, drug manufacturer, device trade name, keyword heading word, floating subheading word, candidate term word] | 3,213 |
| 23 | "POEMS neuropath*".mp. [mp=title, abstract, heading word, drug trade name, original title, device manufacturer, drug manufacturer, device trade name, keyword heading word, floating subheading word, candidate term word] | 17 |
| 24 | "monoclonal gammopath*".mp. [mp=title, abstract, heading word, drug trade name, original title, device manufacturer, drug manufacturer, device trade name, keyword heading word, floating subheading word, candidate term word] | 11,076 |
| 25 | exp polyneuropathy/ | 51,302 |
| 26 | exp acute inflammatory demyelinating polyneuropathy/ | 949 |
| 27 | exp chronic inflammatory demyelinating polyneuropathy/ | 4,944 |
| 28 | familial amyloid polyneuropathy/ | 2,187 |
| 29 | exp diabetic neuropathy/ | 27,898 |
| 30 | exp Guillain Barre syndrome/ | 17,607 |
| 31 | exp paraneoplastic neuropathy/ | 4,601 |
| 32 | exp amyloid neuropathy/ | 1,551 |
| 33 | exp POEMS syndrome/ | 1,668 |
| 34 | 1 or 2 or 3 or 4 or 5 or 6 or 7 or 8 or 9 or 10 or 11 or 12 or 13 or 14 or 15 or 16 or 17 or 18 or 19 or 20 or 21 or 22 or 23 or 24 or 25 or 26 or 27 or 28 or 29 or 30 or 31 or 32 or 33 | 108,632 |
| 35 | "Resistance Training".mp. [mp=title, abstract, heading word, drug trade name, original title, device manufacturer, drug manufacturer, device trade name, keyword heading word, floating subheading word, candidate term word] | 28,886 |
| 36 | "Strength training".mp. [mp=title, abstract, heading word, drug trade name, original title, device manufacturer, drug manufacturer, device trade name, keyword heading word, floating subheading word, candidate term word] | 8,668 |
| 37 | "Strengthening exercise".mp. [mp=title, abstract, heading word, drug trade name, original title, device manufacturer, drug manufacturer, device trade name, keyword heading word, floating subheading word, candidate term word] | 857 |
| 38 | exp resistance training/ | 25,675 |
| 39 | exp functional training/ | 1,444 |
| 40 | exp weight training/ | 396 |
| 41 | exp blood flow restriction training/ | 172 |
| 42 | 35 or 36 or 37 or 38 or 39 or 40 or 41 | 34,431 |
| 43 | 34 and 42 | 180 |

**Supplementary Table 4.** Cinahl search

| **#** | **Cinal search** | **Hits** |
| --- | --- | --- |
| S36 | S30 AND S35 | 194 |
| S35 | S31 OR S32 OR S33 OR S34 | 18,736 |
| S34 | (MM "Resistance Training") | 4,868 |
| S33 | TX "Strengthening exercise" | 955 |
| S32 | TX "Strength training" | 7,86 |
| S31 | TX "Resistance Training" | 12,477 |
| S30 | S1 OR S2 OR S3 OR S4 OR S5 OR S6 OR S7 OR S8 OR S9 OR S10 OR S11 OR S12 OR S13 OR S14 OR S15 OR S16 OR S17 OR S18 OR S19 OR S20 OR S21 OR S22 OR S23 OR S24 OR S25 OR S26 OR S27 OR S28 OR S29 | 40,659 |
| S29 | TX "acute inflammatory demyelinating polyneuropath*" | 123 |
| S28 | TX aidp | 205 |
| S27 | (MM "POEMS Syndrome") | 167 |
| S26 | (MH "Diabetic Neuropathies+") | 15,646 |
| S25 | (MH "Amyloid Neuropathies+") | 462 |
| S24 | (MH "Guillain-Barre Syndrome+") | 1,784 |
| S23 | (MH "Polyneuropathies+") | 9,315 |
| S22 | TX "monoclonal gammopath*" | 1,316 |
| S21 | TX "poems neuropath*" | 5 |
| S20 | TX poems | 8,124 |
| S19 | TX "hereditary neuropath*" | 270 |
| S18 | TX "charcot marie tooth neuropath*" | 103 |
| S17 | TX "Charcot Marie Tooth" | 1,591 |
| S16 | TX "amyloidosis neuropath*" | 2 |
| S15 | TX "diabetes polyneuropath*" | 16 |
| S14 | TX "diabetes neuropath*" | 133 |
| S13 | TX "diabetic polyneuropath*" | 682 |
| S12 | TX "diabetic neuropath*" | 9,03 |
| S11 | TX "non systemic vasculitic neuropath*" | 10 |
| S10 | TX "vasculitic polyneuropath*" | 2 |
| S9 | TX "vasculitic neuropath*" | 109 |
| S8 | TX "vasculitis neuropath*" | 9 |
| S7 | TX "multifocal motor neuropath*" | 285 |
| S6 | TX "guillain barre neuropath*" | 3 |
| S5 | TX "guillain barre syndrom*" | 2,585 |
| S4 | TX "chronic inflammatory demyelinating polyradiculoneuropath*" | 353 |
| S3 | TX "chronic inflammatory demyelinating polyneuropath*" | 675 |
| S2 | TX CIDP | 704 |
| S1 | TX "polyneuropath*" | 5,473  Nederst på formularen |
